# CT of ovarian cancer: 3D cinematic rendering for preoperative evaluation

**DOI:** 10.1186/s13048-018-0461-5

**Published:** 2018-09-26

**Authors:** Yi-ren Jin, Xie-lan Yang, Qin-qing Li, Zhi-ling Yan, Hong-ying Yang, Chengde Liao

**Affiliations:** 1 Radiology Department, Yunnan Cancer Hospital/The Third Affiliated Hospital of Kunming Medical University, 519# Kunzhou Street, Kunming, 650118 Yunnan China; 2 Gynecology Department, Yunnan Cancer Hospital/The Third Affiliated Hospital of Kunming Medical University, 591# Kunzhou Street, Kunming, 650118 Yunnan China

**Keywords:** Ovarian cancer, Computed tomography, X-ray, 3D rendering, Cinematic rendering

## Abstract

**Background:**

Ovarian cancer is the second most common gynecologic malignancy. As the primary imaging modality, computed tomography (CT) can provide staging information for preoperative planning and determination of surgical resectability. As a new three-dimensional postprocessing tool for CT images, cinematic rendering (CR) has the potential to depict anatomic details accurately.

**Case presentation:**

(Case 1) A 44-year-old married woman was diagnosed with recurrent ovarian cancer. CT images indicated the recurrent nodules and masses in the pelvic cavity and the upper middle abdominal peritoneum. The CR image showed that the multiple metastatic lesions and lymph nodes could not be completely removed by reoperation. The patient agreed to receive continued chemotherapy. (Case 2) A 51-year-old woman was admitted to our hospital due to abdominal distension and defecation that had increased for 6 months, with aggravation over the past 3 days. CT examination found cystic and solid masses in the bilateral ovarian area. The CR image demonstrated that the ovarian mass violated the posterior wall of the bladder and the anterior rectal wall. The preoperational imaging evaluation ensured the safety of the operation.

**Conclusion:**

CR could improve the visualization of ovarian cancer masses, metastatic lymph nodes, and peritoneal metastases. CR has a good clinical value and will be more helpful in the preoperational evaluation of ovarian cancer.

## Background

Ovarian cancer is the fifth most common malignancy in women and the second most common gynecologic malignancy [1]. When patients are treated, ovarian cancer is often in the later stage (International Federation of Gynecology and Obstetrics stage III–IV). Surgery is most often performed prior to the initiation of chemotherapy (primary cytoreduction or debulking), and the goal of surgery in this setting is to achieve as complete a gross resection of disease as possible. Meanwhile, the adequacy (optimal vs. suboptimal) of surgical cytoreduction is based on the preoperative evaluation and the maximum diameter of the largest residual tumor after the operation [2]. Locations that need to be carefully evaluated and explored include the right colon, diaphragm, distal part of the great omentum, and para-aortal lymph nodes distal to the left renal vein [2].

Computed tomography (CT) has been established as the primary imaging modality for characterization of ovarian tumors and ovarian cancer staging [3]. Peritoneal metastases ascites, parietal peritoneal thickening or enhancement, and small-bowel wall thickening or distortion can be depicted by cross-sectional imaging [4]. Based on this information, the surgeon will be forewarned of the need for assistance from a gynecologic oncologic surgeon or gastrointestinal oncologic surgeon if a complicated surgical procedure or bowel resection is indicated [3]. In recent years, cinematic rendering (CR), a new technique for postprocessing medical imaging data, has been introduced in preclinical use. Developed by Siemens Healthcare, CR takes into account the complex interaction of photons with the human anatomy and thus generates highly photorealistic images [5]. Bringing an unprecedented degree of realism, CR has begun to be used as a new mean of visualizing vascular structures in regions of complex anatomy [6].

The goal of this study is to demonstrate peritoneal metastases from ovarian cancer using CR. Additional cases are also presented to explore the difficulties of poorly vascularized tumors, which are hardly depicted in frequently used CT visualization methods. This study received approval from the Institutional Review Board of Yunnan Cancer Hospital for a waiver of informed consent, and consent for publication was obtained from the individuals in the presented case reports.

## Case presentation

### Case 1

A 44-year-old married woman (gravida 2) was diagnosed with recurrent ovarian cancer and admitted to our hospital. Five years ago, she underwent concurrent right adnexectomy for the ovarian mass, subtotal hysterectomy, and left adnexectomy. The pathological diagnosis was (right) ovarian serous papillary carcinoma (stage IV). The uterine wall and cervix were affected by cancer tissue, and the left fallopian tube and left ovary were also invaded. She received 12 courses of taxinol plus carboplatin chemotherapy after surgery. Two years prior, a new pelvic mass was found by ultrasound, which was cystic and solid mixed and had a size of about 6 cm × 4.6 cm. Considering the diagnosis of ovarian cancer recurred after chemotherapy, the patient did not continue to receive treatment. Six months ago, CT images showed that multiple nodules and masses in the abdominal cavity and pelvis had increased (10.2 cm × 5.8 cm). Therefore, the patient received two courses of chemotherapy (paclitaxel liposome plus carboplatin) again.

Recently, the patient planned to continue her chemotherapy. The patient’s Karnofsky Performance Scale score was 90, and a hard pelvic mass with unclear boundary could be palpated. The patient’s white blood cell count and hemoglobin level decreased, and degree I chemotherapy-induced myelosuppression was considered.

#### Tumor marker examination

Examination found the carbohydrate antigen 19–9 increased (89.16 kU/L), carcinoembryonic antigen increased (1.57 μg/L), human epididymal protein 4 increased (1477 pmol/L), carbohydrate antigen 153 increased (50.75 kU/L), carbohydrate antigen 125 increased (2210 kU/L). The patient’s premenopausal risk ovarian malignancy algorithm (ROMA) index was 99.72% and postmenopausal ROMA index was 99.46%.

**Fig. 1:**
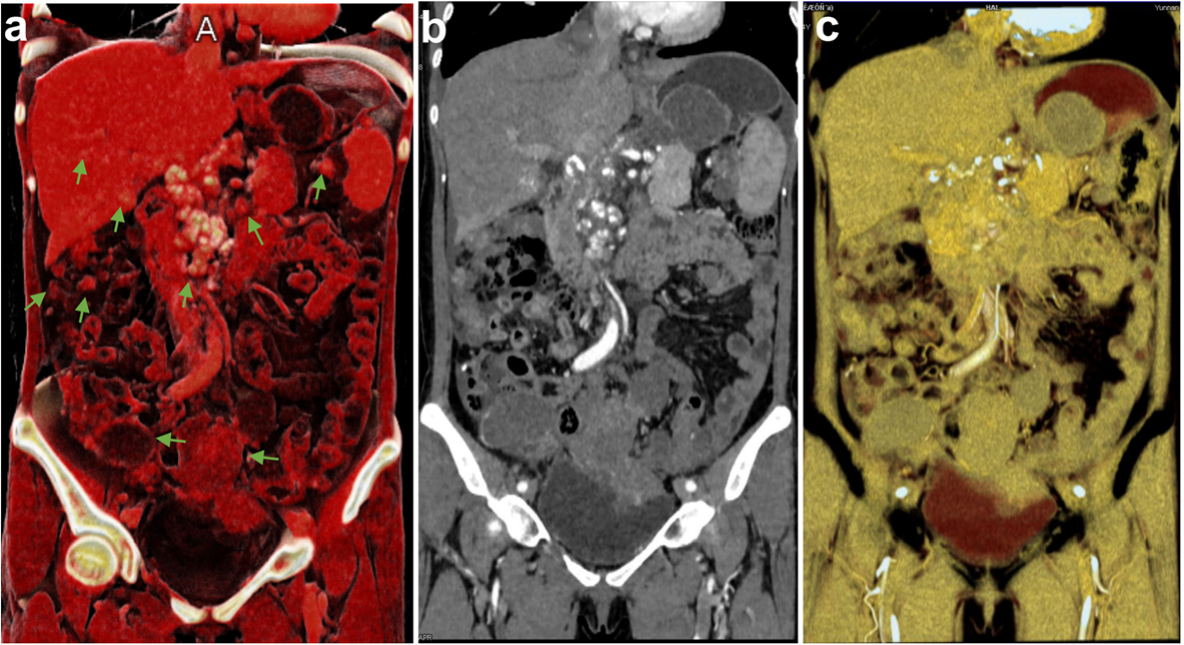
CT images of the abdomen and pelvic cavity in a patient with recurrent ovarian cancer and metastases (case 1). Recurrent nodules and masses in the pelvic cavity, multiple metastatic nodules in the right para colonic sulcus, splenic hilum and hepatic parenchyma, and metastatic lymph nodes in the hepatic portal area and the upper middle abdominal peritoneum. (**a**) CR image showed that the intra-abdominal metastatic nodules had strong stereo effects. The relationship between the lesions and peritoneum and colonic adhesions was clear. Metastatic lymph nodes showed a higher brightness of white because of calcification, while the soft-tissue components around the calcified lesions were also demonstrated clearly. (**b**) The MPR image showed the overall view of the lesions similar to that of CR, but the gray-level image was less detailed and lacked a better texture. The relationship between the metastatic lymph nodes and the adjacent duodenum and the abdominal vessels was not clear. (**c**) Because of the close density between the metastases and the gastrointestinal tract in the VR image, the lesions can hardly be distinguished. The display effect for other structures in the abdominal cavity was also poor

CT showed multiple nodules and masses in the abdominal and pelvic cavity, and some lesions were accompanied by calcification (Fig. 1). The larger mass in the pelvic cavity was 10.2 cm × 5.8 cm in the maximum section image and had an irregular shape and unclear boundaries with the surrounding bowel. Multiple lymph nodes were found on the right axilla, mediastinum, right chest wall, and retroperitoneal space. The right renal pelvis and ureter dilatation (not shown in the figures) was caused by the oppression of swollen lymph nodes. After multidisciplinary team consultation, we considered that the multiple metastatic lesions and lymph nodes could not be completely removed by reoperation. After communicating with the patient and her family, continuation of chemotherapy was agreed upon.

### Case 2

**Fig. 2:**
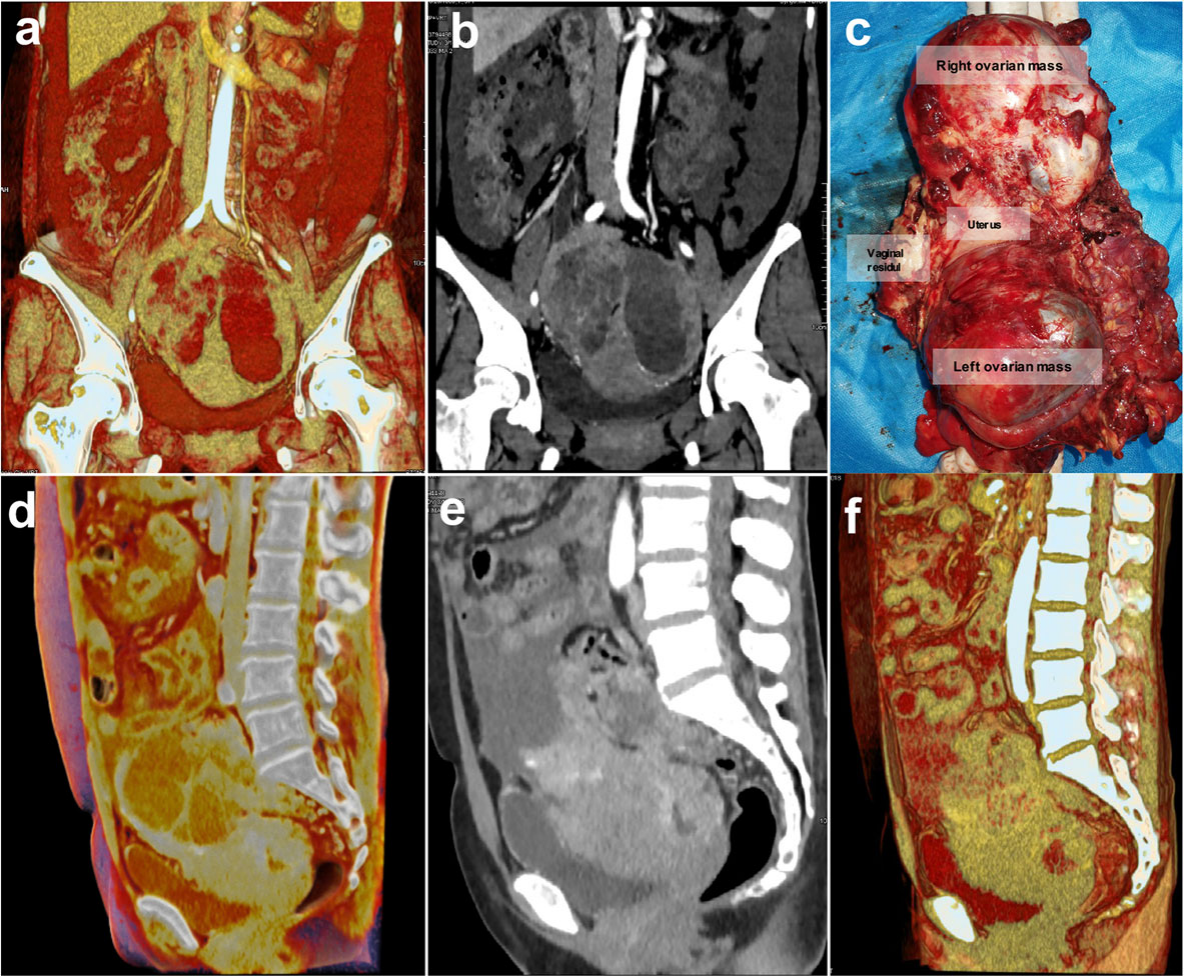
The adhesion of the pelvic mass to the rectum and bladder in CT images and gross pathology (case 2). (**a**-**b**) The large ovarian mass closed the entrance to the pelvis. The lesion was a mixed type of cyst, which was closely connected with the iliac vessels and lateral wall of the pelvic cavity. (**c**) There was no distinct boundary between the mass and the uterus located in the rear. (**a**, **d**) The coronal and sagittal CR image showed that the ovarian mass violated the posterior wall of the bladder and the anterior rectal wall. The normal muscle wall structure of the above cavity viscera disappeared completely. (**b**, **e**) In the MPR image, the boundary of the lesion was displayed because of the ascites and urinary bladder, but the depth of the organ invasion was still difficult to determine. (**f**) VR showed poor effect and could hardly distinguish the lesion from its adjacent organs

A 51-year-old woman (gravida 3) was admitted to our hospital due to abdominal distension and defecation that had increased for 6 months and had been aggravated for 3 days. Six months prior, the patient had abdominal distention, decreased diet, and increased abdominal circumference without obvious inducement. These symptoms were accompanied by lower abdominal discomfort and increased frequency of defecation. Ultrasound examination revealed peritoneal effusion and a pelvic mass that had a maximum cross-sectional area of 14.5 cm × 10.7 cm. Abdominal swelling and a palpable lower abdominal mass with irregular surface and tenderness were found by physical examination. The gynecologist considered that the poor mobility lesion was formed by the uterus and adhesive bilateral ovarian masses, but there were still some gaps between the mass and pelvis wall. The lower margin of the lesion went deeper into the posterior of the uterus and rectum, and no obvious metastatic nodules were found in the vaginal fornix. Premenopausal and postmenopausal ROMA index were increased (99.24% and 99.46%, respectively). Neuron-specific enolase was increased (21.16 μg/L) and sugar antigen-24, − 19, and − 153 were all increased. CT examination found cystic and solid masses (with cystic components) in the bilateral ovarian area (Fig. 2). The peritoneum, greater omentum, and mesentery were thickened and accompanied by multiple nodules. All lesions were contrast enhanced in different degrees, with a large amount of fluid in the pelvic cavity.

During the operation, about 500 mL of red ascites was sucked out, a few miliary nodules on the top of the diaphragm were found, and the liver surface was smooth. The maximal omental mass of about 4 cm in diameter was attached to the spleen. A liver mass of about 6 cm × 5 cm × 3 cm was adhered and extensively infiltrated to the hepatic flexure and ascending colon. The axial diameter of the para-aortic lymph node was about 1.5 cm. There were multiple metastases on the mesentery of 0.5 to 1 cm in length, and the maximum diameter of the bilateral ovarian masses was about 10 cm. The ovarian masses were closely adhered to the sigmoid colon, uterus, and bladder. These masses and adhesions filled and closed the pelvic cavity (Fig. 2a, b, d, e). During the operation, the upper boundary of the lesions reached the pelvic inlet, the lower pole went deeper into the posterior part of the uterus and rectum, its two sides were close to and squeezed the iliac vessels, and the rectum could not be touched. The thickened and hardened posterior wall of the bladder was tightly adhered to the peritoneum, so the mass could not be moved. The appendix was about 8-cm long, which was invaded by the tumor and became thickened and hard. The upper part of the right ureter was thickened, and hydronephrosis was evident with a diameter of 2.5 cm.

The left ovary mass was taken for frozen pathological examination and diagnosed as a malignant ovarian tumor. Then, subtotal ovarian cancer resection and abdominal pelvic tumor cytoreduction were performed (Fig. 2c). The retroperitoneum was removed from the pelvic inlet, and the tumor was then resected from the posterior part of the rectum. The bilateral lower ureter gradually extended to the periphery of the uterus. The whole uterus and bilateral uterine appendages were removed. Resection of the greater omentum and appendix, the sigmoid colon, the rectum, and intestinal anastomosis was performed without visible residual lesions. The operation lasted for 3.5 h. Bleeding during the operation was 1600 mL, infusion was 4250 mL, and transfusion of red blood cells was 4.5 U and of plasma was 600 mL.

## Discussion and conclusions

CR is a new technique for the three-dimensional (3D) visualization of radiologic imaging data such as those provided by CT and magnetic resonance imaging (MRI) [5]. Similar to traditional volume rendering (VR), thin-slice–reconstructed CT data are stacked into a 3D volume. Each isotropic voxel in the volume is assigned a color and transparency based on attenuation thresholds. Unlike VR, which uses a simple ray lighting model, CR uses Monte Carlo path tracing and a global illumination model that takes into account direct and indirect illumination [7]. The CR-generated photorealistic images in these two ovarian cancer cases depicted anatomical structures (including the musculature, bones, and blood vessels) with authentic details, which is more helpful for preoperative surgical and management planning.

Surgery remains the most important facet in the management of ovarian cancer. The purpose of primary cytoreductive surgery is to remove as much tumor as possible in patients with advanced stage (stage III or IV) cancers [8]. The diagnostic laparoscopy was a useful method for assessing the extent of disease in some qualified health centers. But CT and MRI images, including 3D-rendering methods, have long been applied in cases of tumor invasion and preoperational evaluation. In these 2 cases, the metastatic lymph nodes adhered to the abdominal aorta (case 1), and its adhesion to the peritoneum and colon (case 2) helped to differentiate the malignant lesions from benign ones. Meanwhile, CR provides more anatomic structure in detail, and the invasion characteristic of the ovarian lesions was clearly demonstrated.

Based on our initial experience, CR has some potential ways to solve the difficulties of traditional 3D rendering. First, CR could depict the poor natural contrast between metastatic lesions and the gastrointestinal tract, which causes low resolution, complex background, lighting variation, and unrestricted shape and color. Traditional VR and multiplanar reformat (MPR) have no proper template to use when peritoneal metastases are diffused or adhered to the intestinal tube, and the reconstructed images displayed a more confused vision than we expected. However, CR can display the smallest range (within 10 Hounsfield Units) of density differences by setting an exact threshold, just like the metastatic lesions and digestive tract demonstrated different texture in our cases. By considering the direct raying and scattering of objects, CR generated a photorealistic image to display the unresectable metastatic lymph nodes and different features of malignant ovarian masses. Second, CR could, to a certain extent, overcome their lack of showing hypovascular lesions. For hypervascular lesions 3D rendering always obtains satisfactory images, but many peritoneal lesions of ovarian cancer are hypovascular, which is not suitable for traditional VR or MPR. However, CR provides a great degree of operational freedom and an abundant preset template. By careful selection of reconstruction orientation and fine-tuning of parameters, an operator may reveal some features of primary and metastatic ovarian masses (in case 2). Based on a poor lesion-to-background (ascites or bowel) contrast, we could still obtain some satisfactory reconstructed images.

These conveniences may also bring some problems. A disadvantage of CR is operator dependence and variability. We have always obtained a series images with different lighting and color, but some anatomic details were neglected or emphasized by parameter adjustment in our cases. After the CR images and operation records were reviewed by both a gynecologist and a radiologist, we found that some lesions with mimicked intestinal structure could be concealed to an unfamiliar operator. On the contrary, some abdominal structures could display as likely nodules, which might be a pitfall of CR. We are currently conducting a clinical trial to compare all suspected lesions in CR images with their operational and pathological results in ovarian cancer patients. Many similar studies can make a more objective assessment of this new technology.

In our initial experience, CR with highly detailed and lifelike images is very helpful in preoperational evaluation and surgery planning, and there was no extra fee for all 3D rendering in our hospital. The members of our gynecological oncology multidisciplinary team reviewed all patients’ images and discussed the treatment plan specific to each individual patient. Not only the surgeons but also patients and their families were provided useful information from the intuitive CR images. The 3D visualizations can provide a more global impression of the spread of ovarian cancer and allow for high-resolution delineation of the structural changes of adjacent and overlying structures [9]. For patients with advanced ovarian carcinoma, such as the second patient in this report, the scope of the operation and the effect of the resection are within the expected range. The residual lesions were less than 1 cm in maximal diameter, which was considered an optimal surgical result.

Before CR becomes widely used in the evaluation of ovarian cancer, there are still some problems to be solved. First, CR needs to be validated. Created by Siemens Healthcare in 2016, this image-processing software has not been approved for clinical use by the Food and Drug Administration. Although a few scientific papers have discussed the initial experience with CR use and its limitations, ongoing clinical trials will soon help it achieve certification. Second, the efficiency of the algorithm needs to be improved. As of now, the CR software runs on a central server, and it takes seconds or minutes to obtain a reconstruction plane. If you plan a dynamic demonstration with many scripts, other processes on the server will be seriously delayed. The high cost of complete equipment will also become an obstacle to the promotion of this emerging technology.

It is hoped that CR will serve as a useful tool for the preoperative evaluation of ovarian cancer. Broad and prospectively collected clinical data as well as accumulated hands-on experience are needed.

## Abbreviations

3D: Three-dimensional
CR: Cinematic rendering
CT: Computed tomography
MPR: Multiplanar reformat
MRI: Magnetic resonance imaging
ROMA: Risk ovarian malignancy algorithm
VR: Volume rendering
